# Acceptance and Avoidance Processes at Different Levels of Psychological Recovery from Enduring Mental Illness

**DOI:** 10.1155/2015/968596

**Published:** 2015-10-20

**Authors:** Vinicius R. Siqueira, Lindsay G. Oades

**Affiliations:** ^1^School of Psychology, Illawarra Institute for Mental Health, University of Wollongong, Wollongong, NSW, Australia; ^2^Anhanguera, Cascavel, PR, Brazil

## Abstract

*Objective*. This study examined the use of psychological acceptance and experiential avoidance, two key concepts of Acceptance and Commitment Therapy (ACT), in the psychological recovery process of people with enduring mental illness. *Method*. Sixty-seven participants were recruited from the metropolitan, regional, and rural areas of New South Wales, Australia. They all presented some form of chronic mental illness (at least 12 months) as reflected in DSM-IV Axis I diagnostic criteria. The Acceptance and Action Questionnaire (AAQ-19) was used to measure the presence of psychological acceptance and experiential avoidance; the Recovery Assessment Scale (RAS) was used to examine the levels of psychological recovery; and the Scales of Psychological Well-Being was used to observe if there are benefits in utilizing psychological acceptance and experiential avoidance in the recovery process. *Results*. An analysis of objectively quantifiable measures found no clear correlation between the use of psychological acceptance and recovery in mental illness as measured by the RAS. The data, however, showed a relationship between psychological acceptance and some components of recovery, thereby demonstrating its possible value in the recovery process. *Conclusion*. The major contribution of this research was the emerging correlation that was observed between psychological acceptance and positive levels of psychological well-being among individuals with mental illness.

## 1. Introduction

Within the mental health consumer or patient movement, recovery in enduring mental illness does not imply “cure” or remission of illness but the formation of a newly established sense of self based on responsibility and hope for the person's life, suggesting that one should be more optimistic about the future of a person with mental illness [1–3].

Relevant to this recovery process new-generation psychological therapies have been developed. One of these therapies that has shown promising initial results at assisting people who had experienced psychosis is the Acceptance and Commitment Therapy (ACT), to be described in the next section of this paper, which indicated that the possible use of ACT constructs with individuals in recovery from mental illness could help improve their psychological health [4, 5]. Moreover, it is argued that this kind of intervention is consistent with the principles of the recovery model.

Combining the consumer-defined recovery movement with the ACT perspective may prove fruitful. However, recovery and ACT are comprised of too many constructs and variables to be fully covered in this research; therefore the focus of this thesis will follow experiential avoidance and psychological acceptance, important ACT constructs, in the psychological process of recovery from mental illness.

This research focuses on the two main concepts of ACT: experiential avoidance and psychological acceptance. Experiential avoidance has negative effects in one's life [6] and is at the core of several significant clinical problems, such as depression, generalized anxiety disorder, substance abuse, and suicide [7–9]. As such, ACT suggests the use of psychological acceptance to deal with the negative effects of avoidance, which has proven successful at improving the quality of life [10].

Given the pervasiveness of experiential avoidance and the benefits of psychological acceptance, this study has sought to observe whether these two psychological constructs are present in the psychological recovery from mental illness and to examine the part which these two psychological constructs may take in the recovery journey.

## 2. Acceptance and Commitment Therapy

ACT is known as the “third wave” of Behaviour Therapy. The first wave was entitled Radical Behaviourism and focused on the basic learning process of organisms dealing with their environment, where it was seen that the principle under every behaviour was to seek gratification and avoid punishment. The second wave, Cognitive Behaviour Therapy (CBT), emphasized the difference that humans' capacity for language has in their behaviours, providing several benefits but also some disadvantages, which are dealt with in CBT by assisting in effective change of verbal behaviours. ACT, the third wave, is a recent mindfulness-based behaviour therapy that is also concerned about the problems of language but is built under the assumption that the “normal mind” often creates psychological suffering and that attempts to modify this situation will only develop in more clinical distresses. To improve the quality of life, ACT suggests several approaches: psychological flexibility, defusion, acceptance, contact with the present, the observing self, values, and committed action [10–12].

ACT was developed for treatment of a diversity of disorders characterized by experiential avoidance:

*Phenomenon that occurs when a person is unwilling to remain in contact with particular private experiences (e.g., bodily sensations, emotions, thoughts, memories, behavioral predispositions) and takes steps to alter the form or frequency of these events and the contexts that occasion them. ([13, p. 1154])*

Proposing the use of acceptance, the act of “*actively contacting psychological experiences – directly, fully, and without needless defence – while behaving effectively*” [13, p. 1163] to countermeasure experiential avoidance and its negative effects on human life.

## 3. Recovery

Recovery is recognized as a deeply personal and unique process in which a person that deals with an ongoing illness and/or disability searches for purpose and meaning in their lives [3]. This idea is the basis of the recovery movement which advocates for the rights of people living with mental illness to be integrated in today's society and to have the power to determinate their own lives [1].

Other authors such as Andresen et al. [14] and Resnick et al. [15] identify hope as the redefinition of a new sense of self and taking responsibility over one's recovery as key process in the recovery journey.

Recovery is considered a process that does not have a timeline or an end* per se *but is regarded as a continuing process lived by those with mental illness when dealing with the limitations of their psychiatric disability. The evaluations of such criteria are subjective but are linked with objective evaluations of diminishment or withdraw of symptoms, once both aspects are interconnected [16]. It must be noticed that the recovery process should not be confused with rehabilitation as rehabilitation directs itself to practitioners and their methods to facilitate recovery [2].

## 4. Acceptance and Commitment Therapy and Recovery

The literature regarding the use of ACT with people living with mental illness is still in its infancy [4, 5]; however, Siqueira and Oades [17] examined the role and frequency of psychological acceptance and experiential avoidance in the psychological recovery from enduring mental illness. Examples of both were identified in free narratives from people with enduring mental illness describing their experience of recovery.

Siqueira and Oades [17] suggested a likely possible positive relation between the use of psychological acceptance and the onward movement towards recovery. Conversely, they argue that experiential avoidance seemed to indicate setbacks and difficulties when dealing with aspects of mental illness. These initial qualitative results need to be supported by the use of quantitative investigation. Moreover, given the relationship between recovery and psychological well-being, and their combined emphasis on growth, this is also useful to investigate in the context of recovery.

## 5. Psychological Well-Being and Mental Health

Psychological research has indicated that good or positive mental health not only is constituted from the absence of mental illness, as previous paradigms were based on, but also involves factors such as subjective well-being (e.g., happiness and life satisfaction), personal growth (e.g., self-actualization and a sense of meaningfulness), and religiosity (e.g., “other-centeredness” and self-renunciation) [18].

This new paradigm in mental health considers mental illness to be in a different plane rather than being in opposite ends of the same continuum [19]. In this conception, mental health (the same as mental illness) is composed of symptoms that are objectively observed over a period of time and is made up of good or positive mental and social functioning (i.e., subjective well-being) [20].

According to Resnick et al. [21] positive psychology, a movement known for its focus on well-being, and the recovery movement have had parallel tracks, both focusing on the person's strengths and capacities rather than their weaknesses and inabilities.

The recovery movement advocates for the needs of people with mental illness to achieve well-being, empowering them to do so themselves [22], correlating therefore its work with the contemporary view on psychological well-being.

ACT seeks the same objectives within this population, improving one's quality of life (subjective well-being) focusing on the person's values and commitment to “grow.” In this instance, acceptance, rather than avoidance, plays a significant role towards achieving these objectives [4]. Supporting this affirmative, studies with related treatment models such as mindfulness-based approaches showed positive effects in improving the psychological well-being of individuals [23, 24].

## 6. Study Aim

This study sought to examine the relationship between the following variables: (a) psychological acceptance, (b) experiential avoidance, (c) psychological well-being, and (d) recovery for people with enduring mental illness, using the AAQ-19, RAS, and STORI instrument to verify the after-mentioned variables.

## 7. Methodology

### 7.1. Participants

Participants were 41 adults (26 females and 15 males), ranging in age between 21 and 66 years, with a mean age of 42.29 years (SD = 12.83). The participants had been recruited from the metropolitan and rural areas in New South Wales, Australia, and selected on the basis of suitability, as defined by the researcher, based on their demographic characteristics, as well as availability. Participants were included if they had chronic mental illness (at least 12 months) as per DSM-IV provided there was an absence of serious brain injury, intellectual disability, or cognitive disability and if they had greater than five total needs on the Camberwell Assessment of Need (CAN; [25]). The range of existing clinical primary diagnoses reported for the study sample was, with number of participants in parentheses, the following: schizophrenia (5), bipolar disorder (10), major depression (20), posttraumatic stress disorder (3), obsessive compulsive disorder (1), schizoaffective disorder (1), and generalized anxiety disorder (1). The mean length time since the primary diagnosis was given was 10.52 years. Thirty-seven participants reported taking medication.

### 7.2. Procedure

Ethics approval was gained from the relevant university human research ethics committee. Advertisements were run in newsletters, local radios, and printed media, as well as local consumer advocate groups of New South Wales region in Australia. Each participant was screened for suitability by a 10-minute telephone interview and baseline measures were collected before commencement of intervention.

### 7.3. Measures

Four instruments were chosen to verify the occurrence of the variables mentioned in the study aim and subsequently their relations:

1. The AAQ-19 [26] was selected since it is a way of assessing psychological acceptance and experiential avoidance, two key constructs that are at the core of ACT and in this study.
2. The short version of the Recovery Assessment Scale (RAS) [27] was selected to assess the recovery process.
3. The Stages of Recovery Instrument (STORI) (Andresen et al.) [22] was selected since it assesses the psychological recovery from enduring mental illness.
4. The 18-item form of the PWB [19] was selected since it is a way to assess psychological well-being.

## 8. Results

There were no correlations between the overall scores of the RAS instrument and the AAQ-19. The AAQ-19, however, correlated positively with three of the five subscales of the RAS. These were “not dominated by symptoms,” “willingness to ask for help,” and “personal confidence and hope.” The other two subscales (“goal and success orientated” and “rely on others”) had negative correlations.

A correlation between the scores from the AAQ-19 and the Scales of Psychological Well-Being showed positive correlations linking the overall score of psychological well-being with the high use of psychological acceptance, confirming the hypothesis that the use of psychological acceptance is related to improving one's psychological well-being. However, there were no other correlations between the AAQ-19 and the subscales of the Scales of Psychological Well-Being other than the “purpose” subscale.

As it can be seen in Table 3, there is a positive correlation between the scores of RAS and the scores of the Scales of Psychological Well-Being.

## 9. Discussion

From the data collected, 3 hypotheses emerged. The first hypothesis was that there would be significant positive relationship between psychological acceptance and recovery as Table 1 demonstrates there is no relationship between the use of psychological acceptance (as assessed by the AAQ-19) and high recovery (as assessed by the RAS instrument).

Table 1 indicates, however, that some subscales of RAS demonstrated a relationship with psychological acceptance including “willingness to ask for help” which showed a strong relationship with the use of psychological acceptance. Such relationship makes sense since the definition of psychological acceptance as “*actively contacting psychological experiences – directly, fully, and without needless defence – while behaving effectively*” [13, p. 1163] is closely related to willingness, “*the quality or state of being willing; free choice or consent of the will; freedom from reluctance; readiness of the mind to do or forbear*” [28].

The use of psychological acceptance had a positive correlation with the factor “not dominated by symptoms” assessed by the subscale of RAS.

In conclusion, in contrary to prediction, the use of psychological acceptance does not seem to be associated with high levels of recovery from mental illness. However, there was a correlation between three out of the five subscales of RAS and the use of psychological acceptance, demonstrating therefore its possible significance towards important components of the recovery process.

The second hypothesis was that there would be a correlation between psychological acceptance and psychological well-being.

This was confirmed as it can be seen in Table 2 which shows a correlation of the overall score of psychological well-being and the high use of psychological acceptance as assessed by the AAQ-19. However, there were no other significant correlations between the AAQ-19 and the subscales of the Scales of Psychological Well-Being besides the subscale “purpose.” Such result can be explained by the fact that each subscale of the Scales of Psychological Well-Being focuses on detecting specific psychological constructs.

The third hypothesis was that there would be a correlation between the scores of the RAS instrument and the Scales of Psychological Well-Being. As expected there was a positive correlation between the scores of RAS and the scores of the Scales of Psychological Well-Being as shown in Table 3.

### 9.1. Limitations

It must be taken into consideration that this study was limited by the instruments utilized in it. Such instruments were initially chosen by their validity proven in other studies and considered to be the best representative of the psychological constructs studied in this research.

It should be noted that researchers investigating this population in Australia generally experience difficulties with recruiting participants, including finding participants who are available and willing to participate. Nevertheless, a larger sample should be pursued for a follow-up study or one that replicates this study.

A characteristic of the sample used in this study is its diversity of mental health disorders, as defined by the DSM-IV-TR. This research focused on the recovery process of a person dealing with enduring mental illness, in which the type of disorder (e.g., schizophrenia, major depressive disorder, schizoaffective disorder, and bipolar disorder) was not considered to be of relevance due to the similar process that dealing with a mental illness entails, as described in several works, for example, King et al. [2]. However, one could argue that the use of psychological acceptance and experiential avoidance could vary according to the type of mental disorder and as such influence the recovery process. Therefore it is recommended that another study investigates this possibility.

The mean length time since the primary diagnosis of the participants in this study was 10.52 years. Although this characteristic was not taken into consideration given that the participants had volunteered and came from a diverse range of contexts, a study observing the use of psychological acceptance and experiential avoidance in earlier and/or later years since diagnosis from mental illness and its relation with successful recovery from mental illness could prove fruitful.

This characteristic was not taken into consideration in this research given that the participants had volunteered but this variable could play an important role in their answers. However, it must be taken into consideration that even though the development of medication for mental disorders has improved greatly over the years, diminishing the pervasive secondary effects of the drugs, the use of medication can come to interfere with the use of relative complex psychological constructs such as psychological acceptance and experiential avoidance. For this reason, it is recommended that a further study be conducted investigating how this variable could come to affect the use of psychological acceptance and experiential avoidance and its relation with successful recovery from mental illness.

In addition, it should be noted that ACT is not restricted to psychological acceptance and experiential avoidance. There are several other important psychological constructs in ACT, such as defusion, which should be studied in relation to recovery. Such studies are needed since they may reveal valuable data that could help individuals in their recovery process from mental illness. To date there are few publications that link ACT and the recovery model.

## 10. Conclusion

The contribution that this paper has to offer is the emerging relationship that can be seen between the use of psychological acceptance and positive levels of psychological well-being among individuals with mental illness. This represents that psychological acceptance may play a positive role in improving the subjective well-being (e.g., happiness and life satisfaction) of people with mental illness. This may be explained by the fact that psychological acceptance is a fundamental psychological construct used in several mindfulness-based approaches to improve the quality of life of individuals with a chronic illness, such as a mental illness [29].

This research demonstrates, consistent with Siqueira and Oades [17], that recovery is a multifactorial and multidimensional process that incorporates several component processes. One of these processes, hope, may be facilitated by the use of psychological acceptance. Psychological well-being is another dimension in recovery and the use of psychological acceptance may contribute to improving psychological well-being in those who have a diagnosis of mental illness.

## Figures and Tables

**Table 1:** Correlations between the AAQ-19 and the RAS and its subscales.

AAQ-19	RAS (overall score)	Goal and success orientated (RAS subscale)	Not dominated by symptoms (RAS subscale)	Willingness to ask for help (RAS subscale)	Rely on others (RAS subscale)	Personal confidence and hope (RAS subscale)
1.000	.209 (*p* < 0.089)	.143 (*p* < 0.248)	.300^*∗*^ (*p* < 0.014)	.251^*∗*^ (*p* < 0.041)	.067 (*p* < 0.588)	.263^*∗*^ (*p* < 0.032)

^*∗*^Correlation is significant at the 0.05 level.

**Table 2:** Correlations between the AAQ-19 and the Scales of Psychological Well-Being (PWB) and its subscales.

AAQ-19	Psychological Well-Being (PWB overall score)	Acceptance (PWB subscale)	Purpose (PWB subscale)	Mastery (PWB subscale)	Relations (PWB subscale)	Growth (PWB subscale)	Autonomy (PWB subscale)
1.000	−.313^*∗*^	−.192	−.264^*∗*^	−.076	−.132	−.089	−.158
—	.011	.122	032	.544	.289	.479	.206

^*∗*^Correlation is significant at the 0.05 level.

**Table 3:** Correlations between Recovery Assessment Scale (RAS) and Psychological Well-Being (PWB) scales.

RAS	Pearson Correlation	1	−.595^*∗*^
	Sig. (2-tailed)		.000
	*N*	41	41

PWB	Pearson Correlation	−.595^*∗*^	1
	Sig. (2-tailed)	.000	
	*N*	41	41

^*∗*^Correlation is significant at the 0.01 level (2-tailed).
